# Multifocal non-epitheliotrophic cutaneous B-cell Lymphoma in a dog: cytologic, histopathologic, immunophenotypic, and molecular findings

**DOI:** 10.1186/s12917-026-05655-w

**Published:** 2026-06-20

**Authors:** Yunho Jeong, Min-Ji Lee, Sooyoung Choi, Dae-Yong Kim, Jin-Young Chung, Jin-Ok Ahn, Eun Wha Choi

**Affiliations:** 1 Department of Veterinary Internal Medicine, College of Veterinary Medicine, Kangwon National University, 1 Kangwondaehak-gil, Chuncheon, 24341 Gangwon-do Republic of Korea; 2 Haemaru Companion Animal Clinical Research Institute, 8-4, Hwangsaeul- ro 319beon-gil, Bundang-gu, Seongnam-si, 13590 Gyeonggi-do Republic of Korea; 3 Department of Veterinary Medical Imaging, College of Veterinary Medicine, Institute of Veterinary Science, Kangwon National University, 1 Kangwondaehak-gil, Chuncheon, 24341 Gangwon-do Republic of Korea; 4 Department of Veterinary Pathology and Research Institute of Science, College of Veterinary Medicine, Seoul National University, 599 Gwanak-ro, Gwanak-gu, Seoul, 08826 Republic of Korea; 5 Department of Veterinary Clinical Pathology, College of Veterinary Medicine, Institute of Veterinary Science, Kangwon National University, 1 Kangwondaehak-gil, Chuncheon, 24341 Gangwon-do Republic of Korea

**Keywords:** Canine, Cutaneous B-cell lymphoma, Immunohistochemistry, Flow cytometry, Polymerase chain reaction for antigen receptor rearrangement, CD79a, PAX5, Ki-67, MUM-1

## Abstract

**Background:**

Cutaneous B-cell lymphoma is extremely rare in dogs; only few cases have been reported in veterinary literature, and its clinicopathological characteristics, biological behavior, and classification remain incompletely characterized.

**Case presentation:**

A 12-year-old spayed female Shih Tzu presented with extensive, multifocal subcutaneous nodules. Histopathology revealed infiltration of neoplastic lymphoid cells within the dermis and subcutaneous tissue without epidermal involvement. Immunohistochemistry revealed strong positivity for cluster of differentiation (CD)79a and paired box 5 (PAX5), no CD3 expression, strong nuclear expression of multiple myeloma oncogene 1 (MUM-1), and a relatively low Ki-67 labeling index (14.28%), suggesting limited proliferative activity. Flow cytometric analysis confirmed B cell dominance, and polymerase chain reaction for antigen receptor rearrangement (PARR) demonstrated clonal rearrangement of the immunoglobulin heavy chain gene. Cytological evaluation of the liver and spleen did not reveal definitive evidence of lymphoma infiltration, and PARR analysis of the splenic aspirate showed polyclonal lymphoid population, providing no molecular evidence of clonal lymphoid proliferation. Despite treatment with cyclophosphamide, doxorubicin, vincristine, and prednisolone (CHOP)-based chemotherapy, only a transient partial response was observed, followed by rapid disease progression.

**Conclusion:**

This case report describes a rare multifocal non-epitheliotrophic cutaneous B-cell lymphoma in a dog, characterized by aggressive biological behavior, high proliferative activity, and poor response to conventional chemotherapy. These findings highlight the need for further studies to better define the clinicopathological spectrum and optimal management of canine cutaneous B-cell lymphoma.

**Supplementary Information:**

The online version contains supplementary material available at https://doi.org/10.1186/s12917-026-05655-w.

## Background

 Canine lymphoma is a common hematopoietic neoplasm in dogs that is characterized by marked heterogeneity in anatomical distribution, histological features, and immunophenotypes, and multicentric disease is the most prevalent form [1]. According to the World Health Organization (WHO) classification system, canine lymphomas are categorized based on their anatomical location, morphological features, and immunophenotypic characteristics, which collectively guide diagnostic and prognostic evaluations [2].

Cutaneous lymphoma is an uncommon extranodal manifestation of canine lymphoma that may present with a broad spectrum of clinical features. Canine cutaneous T-cell lymphoma most commonly presents as an epitheliotropic form characterized by multifocal erythematous, alopecic, and exfoliative dermatitis that may progress to plaques and nodules, whereas non-epitheliotropic variants typically manifest as dermal or subcutaneous nodules with less prominent epidermal involvement [3]. Canine cutaneous lymphoma is predominantly of T-cell lineage with B-cell phenotype being exceedingly rare [4, 5].

Consequently, the biological behavior and prognostic implications of cutaneous B-cell lymphoma remains incompletely characterized. This report describes a dog with multifocal cutaneous B-cell lymphoma and provides integrated clinical, histopathological, immunophenotypic, and molecular findings to further contribute to our understanding of this rare entity.

## Case presentation

**Fig. 1:**
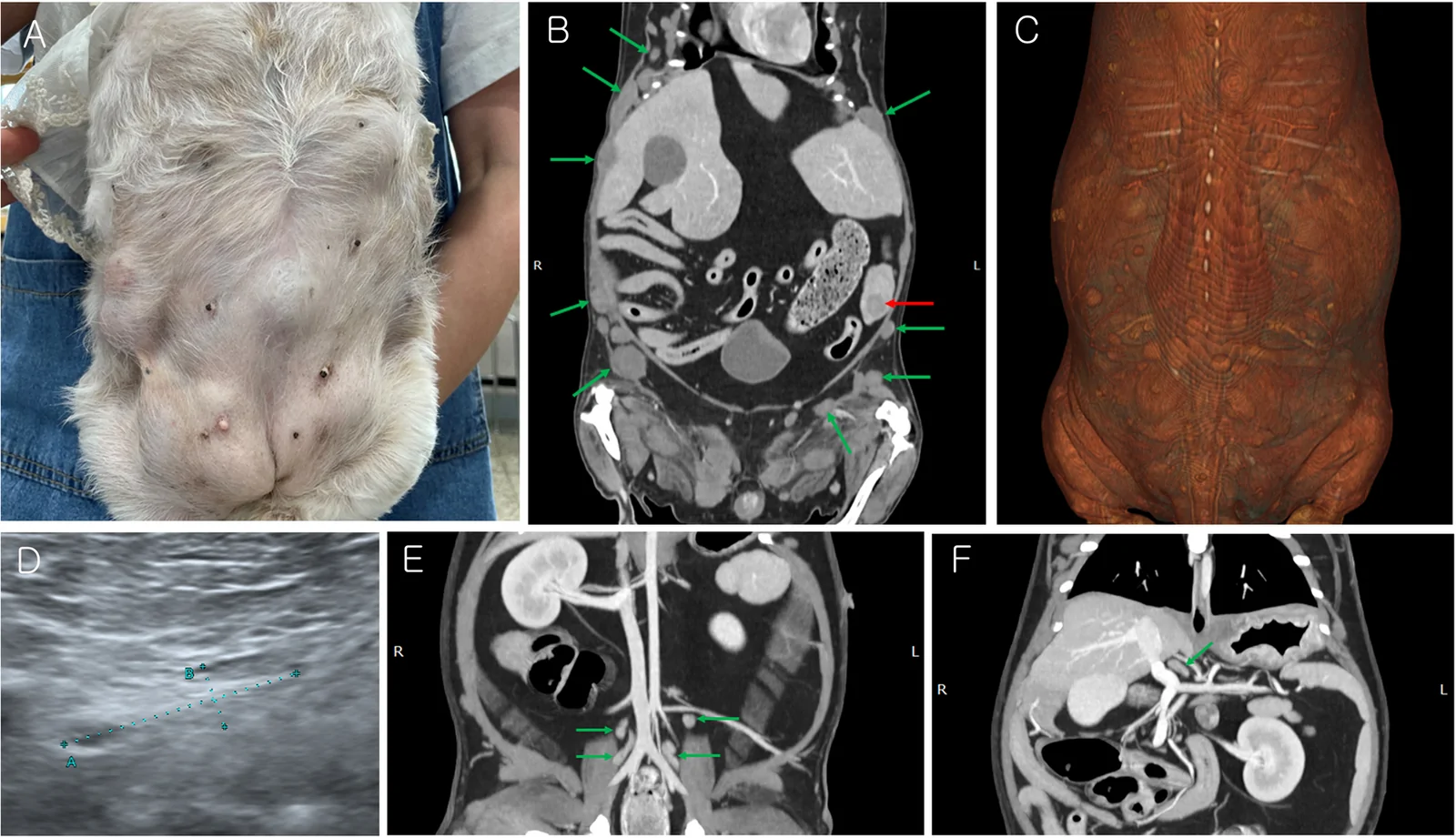
Cutaneous lesions and imaging findings. **A** Cutaneous lesions prior to initiating multidrug chemotherapy. Multiple raised cutaneous nodules causing irregular, nodular contour of the abdominal skin without ulceration. **B** Coronal reconstructed CT image showing diffusely distributed subcutaneous soft tissue nodules (green arrows) consistent with multifocal cutaneous involvement. A splenic nodule is also noted (red arrow), suggesting lymphomatous involvement, although benign and non-neoplastic processes, such as nodular hyperplasia and extramedullary hematopoiesis, are also probable. **C** Three-dimensional volume-rendered CT image demonstrating the distribution of subcutaneous nodules across the dorsal abdomen. **D** Ultrasonographic image showing mild enlargement of the left medial iliac lymph node (14.5 × 3.8 mm). **E** Contrast-enhanced coronal reconstructed CT image showing mildly enlarged sublumbar lymph node centers with minimal changes (green arrows). **F** Contrast-enhanced transverse CT image demonstrating mildly enlarged hepatic lymph nodes (8.31 × 9.5 mm, green arrow)

A 12-year-old spayed female Shih Tzu presented with a 3-month history of extensive multifocal cutaneous nodules without other dermatologic signs such as ulceration, alopecia, erythema, or pruritus. On physical examination, multiple firm and slightly movable subcutaneous masses were noted, predominantly on the ventral abdomen, with intact overlying skin (Fig. 1A).

Complete blood count revealed no significant abnormalities. Serum biochemistry demonstrated elevated liver enzyme activities (alanine aminotransferase [ALT], 102 U/L; reference range, 19–70 U/L; alkaline phosphatase [ALP], 2112 U/L; reference range, 15–127 U/L; gamma-glutamyl transferase [GGT], 17 U/L; reference range, 0–6 U/L), increased blood urea nitrogen (BUN, 38.4 mg/dL; reference range, 8–26 mg/dL), and hypertriglyceridemia (330 mg/dL; reference range, 19–133 mg/dL).

**Fig. 2:**
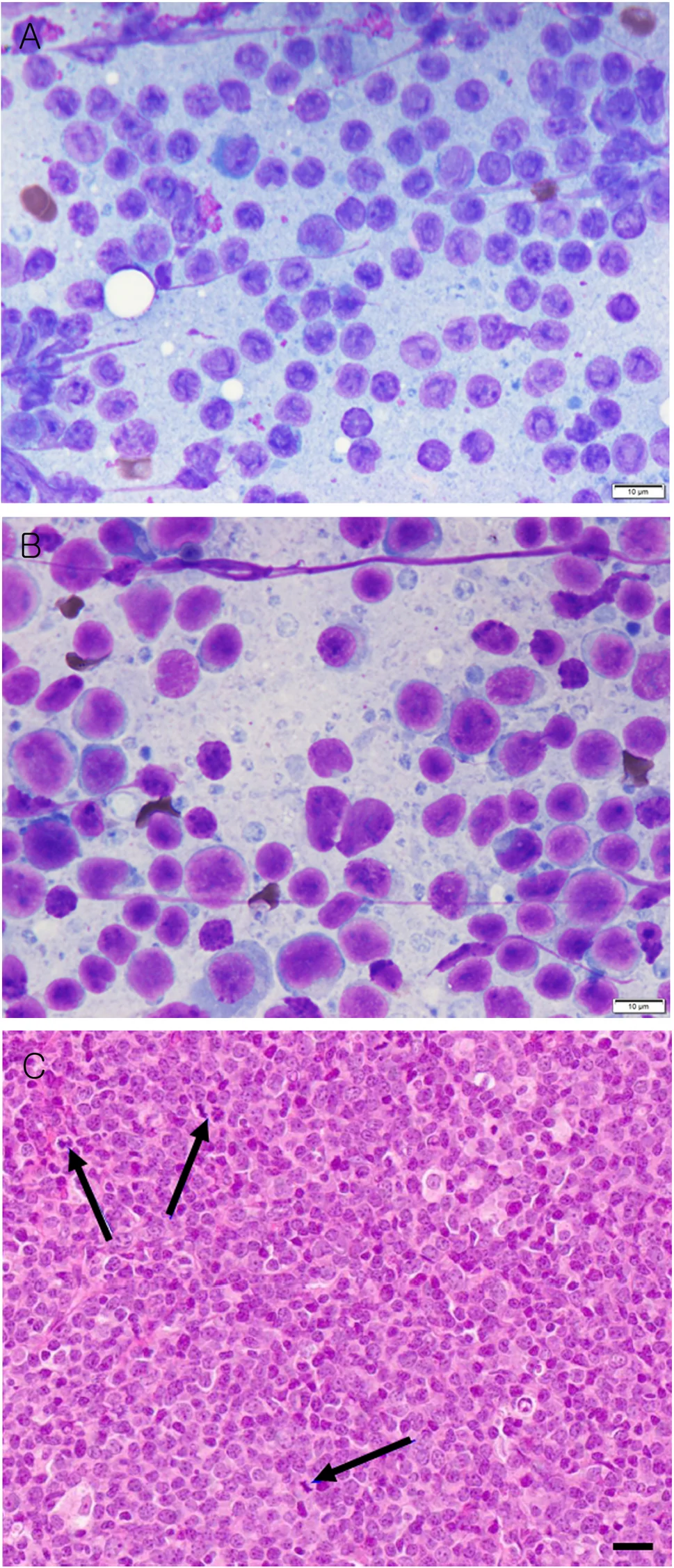
Cytologic and histopathologic findings of the cutaneous mass. **A **Cytologic examination of fine-needle aspirates from the cutaneous masses revealed numerous lymphoglandular bodies and a predominance of small- to medium-sized lymphoid cells. In the context of the gross and imaging findings, these cytologic features were considered suggestive of cutaneous lymphoma based on cytologic features (Diff-quick stain ×1000, Scale bar = 10 μm). **B** Impression smear of the cutaneous mass, revealing similar cytologic features to those observed on fine-needle aspiration (Diff-quick stain ×1000, Scale bar = 10 μm). **C** Histopathologic section of the cutaneous mass demonstrating diffuse infiltration of numerous round neoplastic cells throughout the dermis. These round cells were small- to medium-sized neoplastic lymphoid cells characterized by sharply demarcated nuclei, prominent one to two nucleoli, scant cytoplasm, and a non-cleaved nuclear appearance. Occasional mitotic figures were observed (arrows; 0–3 per ×400 high-power field). (H&E, ×400, Scale bar = 20 μm.)

Computed tomography and ultrasonography revealed diffuse subcutaneous soft tissue nodules (Fig. 1B and C), mild enlargement of abdominal lymph nodes with minimal changes and uncertain clinical significance (Fig. 1D–F), and a focal splenic lesion (Fig. 1B). Given the clinical context, the splenic nodule raised concerns of lymphomatous involvement, although, benign and non-neoplastic processes, including nodular hyperplasia and extramedullary hematopoiesis, were also considered differential diagnoses. Fine-needle aspiration of the cutaneous masses revealed numerous lymphoglandular bodies and a population of predominantly small-to-medium-sized round lymphoid cells. Considering the gross and imaging findings, these cytological features were suspicious for lymphoma (Fig. 2A).

Following the identification of hepatomegaly in imaging studies, fine-needle aspiration of the liver was performed for further evaluation. Rare atypical lymphoid cells were observed in the hepatic fine-needle aspirate; however, given their extremely low frequency, these findings were considered suggestive, but not diagnostic, of hepatic involvement (Supplementary Fig. 1A). Fine-needle aspiration of the splenic nodule was markedly limited by blood contamination. The smear contained numerous small-to-medium-sized lymphoid cells, interpreted as background lymphoid cells, with round to mildly indented nuclei and moderately condensed chromatin. A subset of lymphoid cells exhibited relatively abundant pale basophilic cytoplasm, and occasional binucleated lymphoid cells were observed. Importantly, no cytological features suggestive of primary splenic neoplasms or metastatic tumors of non-lymphoid origin were observed. In other areas of the smear, megakaryocytes and nucleated erythrocytes (erythroid precursors) were also identified, consistent with splenic extramedullary hematopoiesis (Supplementary Fig. 1B and C). However, owing to marked blood contamination and the absence of definitive cytologic criteria for malignancy, splenic involvement in lymphoma could not be cytologically confirmed. To further evaluate clonality, polymerase chain reaction for antigen receptor rearrangement (PARR) analysis was performed on the DNA extracted from the splenic aspirate; however, the results demonstrated a polyclonal lymphoid population.

For definitive diagnosis, a 3-mm punch biopsy of a representative cutaneous lesion was performed. The cytology of the cutaneous masses obtained by impression is shown in Fig. 2B and is consistent with the findings of fine-needle aspiration. Histopathological examinations and immunohistochemistry were performed by two diplomats at the Korean College of Veterinary Pathologists in a commercial veterinary diagnostic pathology service laboratory (PATH, Seoul, Republic of Korea). Histopathological examination revealed dense infiltration of round neoplastic cells within the dermis and subcutaneous tissue. (Fig. 2C). Representative histopathological features of the cutaneous mass at various magnifications are presented in Supplementary Fig. 2, including a low-power view showing a dermal/subcutaneous mass separated from the epidermis without evidence of epitheliotropism, thus supporting a non-epitheliotropic pattern.

Immunohistochemistry was performed on 4-µm-thick sections of formalin-fixed, paraffin-embedded tissue samples. The sections were deparaffinized in xylene and rehydrated using graded ethanol. Antigen retrieval was performed using heat-induced epitope retrieval in citrate buffer (pH 6.0) or EDTA buffer (pH 9.0), according to the manufacturer’s recommendations. Endogenous peroxidase activity was blocked using 3% hydrogen peroxide. The sections were incubated with primary antibodies against CD3, CD79a, PAX5, MUM-1, and Ki-67 at 4 °C for 24 h. All antibodies used in this study were previously validated for diagnostic immunohistochemistry. The antibody clones, dilutions, and vendors are summarized in Table 1. Immunoreactivity was detected using a polymer-based horseradish peroxidase detection system with 3,3′-diaminobenzidine as the chromogen, followed by hematoxylin counterstaining. Appropriate positive and negative controls were included in each staining run. Immunoreactivity was evaluated based on the presence, distribution, and intensity of positive staining in the neoplastic and non-neoplastic lymphoid cells. Ki-67 expression was assessed by manually counting Ki-67-immunopositive neoplastic cells in 10 high-power fields (HPFs; 400× magnification) of the immunostained sections. The percentage of Ki-67-positive cells was calculated relative to the total number of neoplastic cells in each field, and the mean value was used as the Ki-67 labeling index.

**Table 1:** Primary antibodies used for immunohistochemical staining of a dermal mass

Antibody	Host	Source	Clone/ID	Clonality	Dilution	Antigen retrieval	Positive control
CD3	Rabbit	Dako A0452	UCHT1	pAb	1:200	Citrate	Lymph node
CD79a	Mouse	Dako M7051	HM57	mAb	1:100	Citrate	Lymph node
PAX5	Mouse	BD Biosciences 610,863	24/Pax-5	mAb	1:50	Citrate	Lymph node
Ki-67	Mouse	Dako M7240	MlB-1	mAb	1:50	Citrate	Mast cell tumor
MUM-1	Mouse	Dako M7259	MUM1p	mAb	1:20	Tris-EDTA	Plasmacytoma

*CD* Cluster of Differentiation, *PAX5* Paired Box 5, *MUM-1* Multiple Myeloma Oncogene 1, *pAb* polyclonal antibody, *mAb* monoclonal antibody

**Fig. 3:**
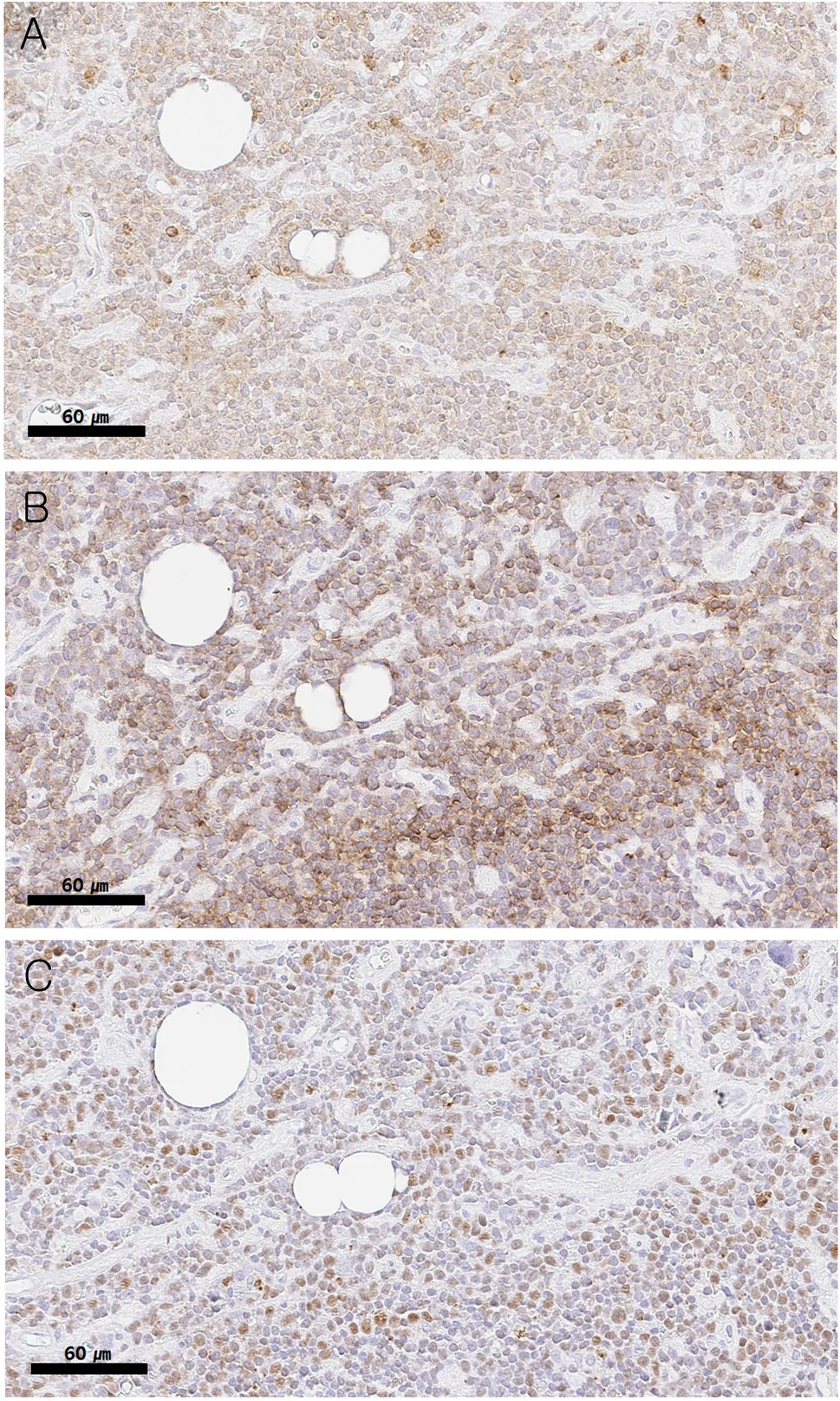
Immunohistochemical characterization of cutaneous lymphoma. **A **CD3 immunostaining demonstrating rare scattered T cells, which are identified as a small population of cells showing strong brown cytoplasmic/membranous labeling. **B** Diffuse cytoplasmic positivity for CD79a in neoplastic round cells. **C** Strong and diffuse nuclear positivity for PAX5 in most neoplastic cells. (Scale bars = 60 μm)

**Fig. 4:**
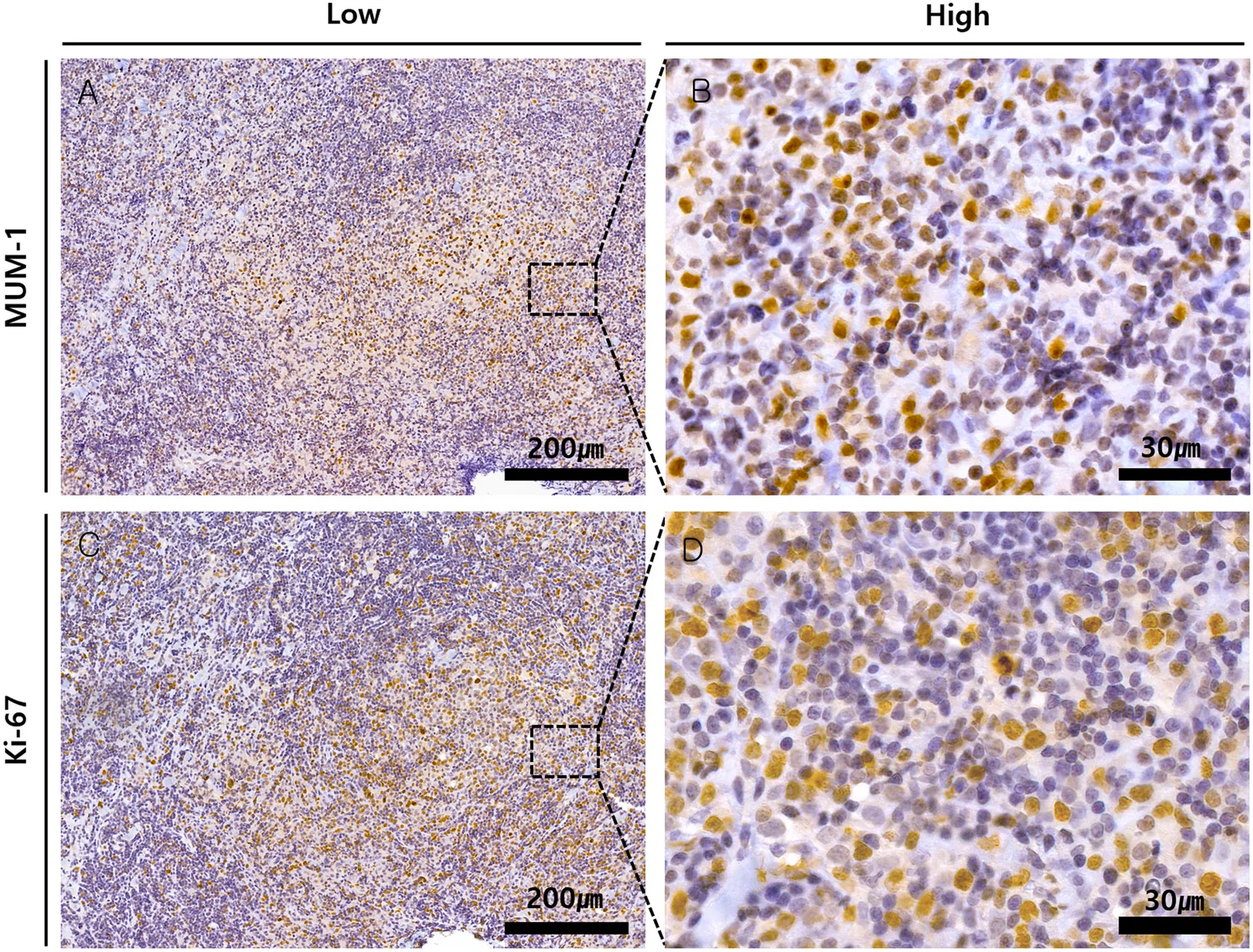
Immunohistochemical staining for MUM-1 and Ki-67 in neoplastic lymphoid cells. **A** Low-power view of MUM-1 immunostaining showing multifocal nuclear positivity in neoplastic cells. **B** High-power view of MUM-1 immunostaining demonstrating strong nuclear positivity in many neoplastic cells, supporting an activated B-cell or post–germinal center phenotype. **C** Low-power view of Ki-67 immunostaining showing scattered nuclear positivity among neoplastic cells. **D** High-power view of Ki-67 immunostaining demonstrating a relatively low proliferative index, with approximately 14.28% of neoplastic cells showing nuclear positivity. (Scale bars = 200 μm in A and C; 30 μm in B and D.)

**Fig. 5:**
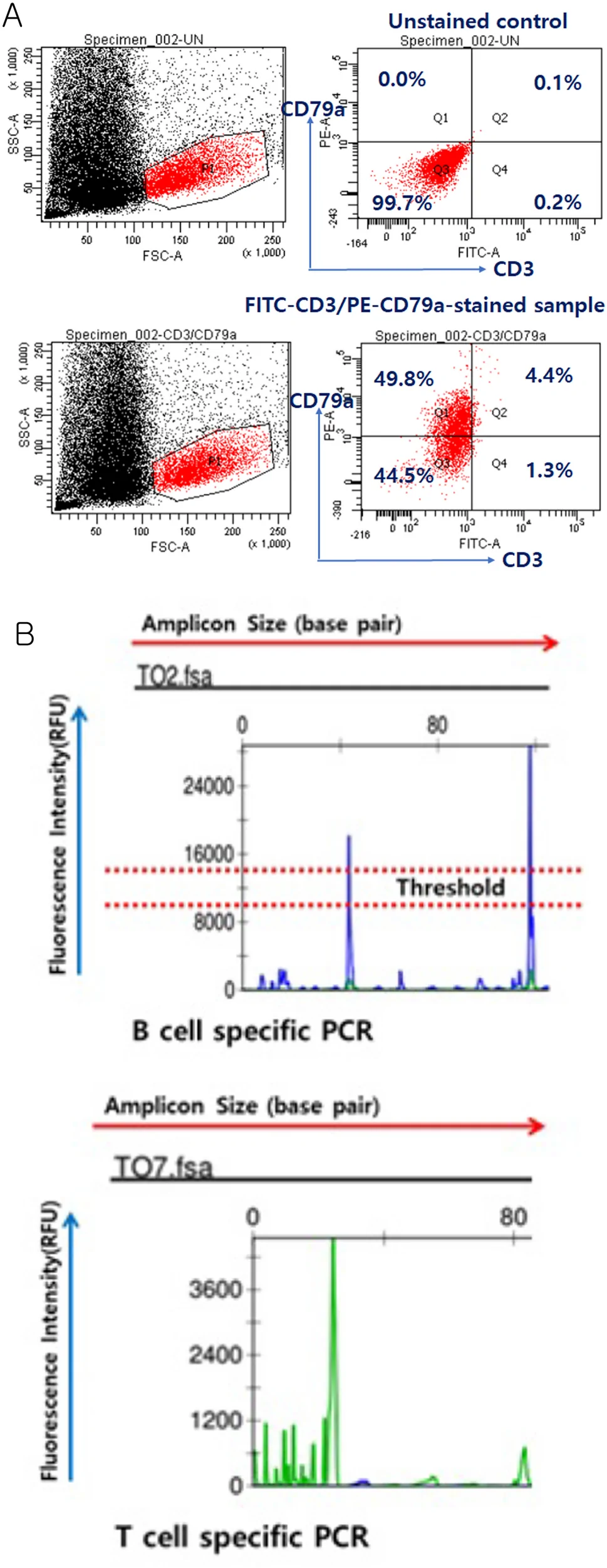
Flow cytometric and molecular analysis of the cutaneous lesion. **A** Flow cytometric analysis of cells obtained from fine-needle aspirates of the cutaneous lesion. The unstained control is shown in the upper panels, and the FITC-CD3/PE-CD79a–stained sample is shown in the lower panels. Gating on the lymphoid population showed that CD79a-positive B cells were predominant within the lymphoid population, with few CD3-positive T cells. **B** PCR for antigen receptor rearrangement (PARR) analysis performed using DNA obtained from a fine-needle aspiration (FNA) slide from the ventral cutaneous lesion. In the microcapillary electrophoresis results, the x-axis represents the size of amplified PCR products in base pairs (bp), whereas the y-axis represents relative fluorescence units (RFU). The expected target sizes were approximately 120 bp for immunoglobulin heavy chain (IgH) gene rearrangement and 90 bp for T-cell receptor (TCR) gene rearrangement. Clonality was assessed based on the presence of a distinct dominant peak within the expected target size range. B-cell–specific PCR targeting immunoglobulin heavy chain (IgH) gene rearrangement demonstrated a distinct dominant clonal peak, whereas T-cell receptor gene rearrangement showed a polyclonal pattern, consistent with clonal B-cell expansion

Immunohistochemical staining demonstrated strong and diffuse positivity for CD79a and PAX5, whereas CD3 staining was negative, confirming B-cell lineage (Fig. 3A–C). Approximately 3%, 90%, and 80% of the examined cells were positive for CD3, CD79a, and PAX5, respectively. Additional immunohistochemical evaluation revealed strong nuclear immunoreactivity for MUM-1 in approximately 80% of the neoplastic round cells, supporting an activated B-cell or post–germinal center phenotype (Fig. 4A and B). Furthermore, the Ki-67 labeling index was 14.28%, suggesting relatively limited proliferative activity (Fig. 4C and D). Flow cytometry analysis was performed on a cell suspension obtained from fine-needle aspirates of the cutaneous lesion using a previously described method [6]. The analysis revealed a predominance of CD79a-positive B cells with few CD3-positive T cells, supporting a B-cell-dominant lymphoid population (Fig. 5A). To further assess clonality, a cutaneous lesion sample, specifically, a fine-needle aspiration (FNA) slide obtained from the ventral cutaneous lesion, was submitted to a reference laboratory (GREEN VET, Yongin, Republic of Korea) for real-time PCR–based PARR testing. The amplified PCR products were analyzed using capillary electrophoresis for clonality. Detailed assay conditions and interpretation criteria for PARR have been described previously [6]. B-cell-specific PCR targeting immunoglobulin heavy chain (IgH) gene rearrangement revealed a distinct dominant clonal peak, whereas T cell receptor gene rearrangement showed a polyclonal pattern (Fig. 5B). These findings confirmed clonal B-cell proliferation and supported cutaneous B-cell lymphoma diagnosis.

A modified University of Wisconsin 19-week cyclophosphamide, doxorubicin, vincristine, and prednisolone (CHOP)-based chemotherapy protocol was initiated. The dog initially showed a partial clinical response to the first treatment. However, chemotherapy administration was repeatedly delayed owing to adverse events and concurrent complications, including sialocele rupture and pneumonia associated with chemotherapy-induced neutropenia. After two treatment cycles, the disease still progressed, with a progression-free interval (PFI) of 84 days, and the owner declined further treatment. The patient died of respiratory distress approximately 1 month after the discontinuation of chemotherapy and approximately 6 months (180 days) after the diagnosis of cutaneous lymphoma. A necropsy was not performed because the owner declined postmortem examination.

## Discussion and conclusions

Cutaneous lymphoma is a rare form of cutaneous lymphoid neoplasia in dogs, of predominantly T cell origin. Histologically, cutaneous lymphomas in dogs are broadly classified into epitheliotropic and non-epitheliotropic forms, based on the presence or absence of epidermal infiltration by neoplastic lymphocytes [2]. Epitheliotropic cutaneous lymphoma predominantly involves the epidermis and adnexal epithelium and is the most frequently diagnosed form, whereas non-epitheliotropic cutaneous lymphoma is considerably less common and is characterized by the infiltration of neoplastic lymphoid cells within the dermis and/or subcutaneous tissue without prominent epidermotropism. Epitheliotropic lymphoma is consistently of T-cell origin. In contrast, non-epitheliotropic lymphoma is predominantly derived from T cells, although B cell origin has also been reported in a subset of cases [7]. In this case, the neoplastic infiltrate was confined to the cutis and subcutis. Although the epidermis was separated from the underlying dermis and subcutis in the biopsy specimens, there was no evidence of neoplastic infiltration of the epidermis. Therefore, an epitheliotropic pattern was excluded, and the lesion was considered consistent with non-epitheliotropic cutaneous lymphoma.

Non-epitheliotropic cutaneous B-cell lymphoma is exceedingly uncommon, with only sporadic cases described in the veterinary literature [2]. Among these, a Golden Retriever developed slowly progressive small nodular lesions distributed over the interscapular region, abdomen, groin, thighs, and axillae without evidence of systemic involvement [4]. Additionally, a 12-year-old Beagle presented with multiple cutaneous and subcutaneous nodules; however, the pathological findings were inconclusive, precluding definitive differentiation between plasmablastic B-cell lymphoma and cutaneous plasmacytosis [5]. Further supporting the morphological diversity of this entity, a rare case of cutaneous non-epitheliotropic B-cell lymphoma with atypical spindle cell morphology was reported in a Weimaraner dog, highlighting that neoplastic B cells may occasionally exhibit marked cytological pleomorphism that mimics mesenchymal tumors [8]. In addition, subcutaneous B-cell lymphomas presenting as multiple dermal and subcutaneous nodules with favorable long-term outcomes after surgical excision and chemotherapy have also been documented, emphasizing that B-cell lymphomas can present as localized cutaneous/subcutaneous masses with variable clinical behavior [9]. Similarly, the present case showed no evidence of systemic involvement; however, it manifested as extensive multifocal cutaneous and subcutaneous lesions involving both the dorsal and ventral trunks, forming a confluent mass-like lesion with an unusual clinical presentation. Although cutaneous and subcutaneous B-cell lymphomas in dogs have been reported to exhibit a broad clinical spectrum, including localized nodular disease, morphological variability, and variable clinical outcomes, the present case was characterized by an aggressive clinical course, including an incomplete response to chemotherapy and rapid disease progression.

The clinicopathological abnormalities observed in this case were most likely attributable to concurrent pituitary-dependent hyperadrenocorticism rather than the cutaneous lymphoma itself. Marked elevation of serum ALP, together with increased BUN and hypertriglyceridemia, is consistent with the metabolic and hepatocellular changes commonly associated with hyperadrenocorticism in dogs. Therefore, these biochemical alterations are considered non-specific and unrelated to the primary neoplastic process.

Cutaneous lymphoma in dogs may occur as a primary skin disease or as part of systemic lymphoma with secondary cutaneous involvement. According to the World Health Organization (WHO) classification and veterinary oncology literature, primary cutaneous lymphoma is defined as a lymphoma confined to the skin at the time of diagnosis, without evidence of systemic or extracutaneous organ involvement, whereas secondary cutaneous lymphoma reflects skin infiltration by disseminated systemic lymphoma [2]. Distinguishing between primary cutaneous lymphoma and secondary cutaneous involvement in systemic lymphoma can be challenging without comprehensive clinical evaluation, staging work-up, and histopathological assessment, highlighting the heterogeneity of cutaneous B-cell lymphomas in dogs. This underscores the importance of a comprehensive diagnostic approach that integrates clinical findings, histopathology, immunophenotyping, and molecular analyses for accurate prognosis and therapeutic decision making. In this case, a comprehensive staging work-up, including imaging, cytology of the abdominal organs, and molecular clonality assessment, did not provide convincing evidence of systemic lymphoma. No clinically significant lymphadenomegaly or organ involvement suggestive of disseminated disease was identified on imaging. Cytological evaluation of the liver and spleen revealed rare atypical lymphoid cells without definitive evidence of clonal infiltration. In addition, PARR analysis of splenic aspirates revealed a polyclonal lymphoid population, further supporting the absence of detectable systemic neoplastic expansion. However, definitive histopathological evaluation of the regional lymph nodes was not performed, and postmortem examination was unavailable. Therefore, the possibility of subclinical or early systemic involvement cannot be completely excluded.

Immunophenotyping is essential for accurate lineage determination, as morphological features alone cannot reliably distinguish B-cell from T-cell origin [10]. In the present case, strong and diffuse immunoreactivity for CD79a and PAX5 along with no CD3 expression confirmed B-cell lineage and supported cutaneous B-cell lymphoma diagnosis.

Further immunophenotypic characterization revealed strong nuclear expression of MUM-1 in a large proportion of neoplastic cells, suggesting an activated or post–germinal center B-cell phenotype. MUM-1 expression is associated with activated B cell differentiation and plasmablastic features in canine lymphomas, although its prognostic significance remains unclear in veterinary oncology [11, 12]. However, in human lymphomas, MUM-1 expression is a well-established marker of biologically aggressive diseases. In follicular lymphoma, MUM-1 expression dichotomizes cases into MUM-1–positive high-grade and MUM-1–negative low-grade forms [13]. Moreover, in diffuse large B-cell lymphoma (DLBCL), MUM-1 is strongly associated with the activated B-cell-like (ABC) subtype, which is consistently linked to worse prognosis compared to germinal center B-cell-like subtype. In primary cutaneous B-cell lymphomas, abnormal MUM-1 expression is associated with activated B-cell phenotype and adverse clinical behavior, particularly in leg type primary cutaneous diffuse large B-cell lymphoma [14]. In a recent pilot study evaluating canine lymphomas, MUM-1 expression was more frequently observed in high-grade B-cell lymphomas and was proposed to reflect the biologically aggressive behavior associated with activated B-cell differentiation [11]. Furthermore, MUM-1/IRF4 immunoreactivity has been documented in canine plasmacytomas and other round cell neoplasms, supporting its role as a marker of late-stage B cell differentiation and plasmacytic activation [12]. Compared with previously reported cases of canine cutaneous B-cell lymphoma, immunophenotypic characterization, including MUM-1 expression, has rarely been described in detail, limiting direct comparison. Nevertheless, although direct extrapolation from human to canine lymphoma should be performed with caution, a recent pilot study on canine lymphoma suggested that MUM-1 expression might be associated with biologically aggressive behavior [11]. Therefore, the strong and diffuse MUM-1 expression observed in the present case may be consistent with an aggressive biological phenotype and may partially explain the rapid disease progression and poor therapeutic response observed clinically.

Ki-67 immunostaining was used to assess proliferative activity, as increased Ki-67 expression has been associated with aggressive biological behavior and poorer clinical outcomes in some canine lymphoma subtypes [15, 16]. In addition, flow cytometric assessment of Ki-67 expression has been shown to be a reliable method for evaluating proliferative activity in canine lymphoid populations [17]. However, the prognostic significance of Ki-67 expression in canine lymphoma remains controversial, and no universally accepted cutoff value has been established. Previous studies have reported variable associations between the Ki-67 index and clinical outcomes, depending on the lymphoma subtype, treatment protocol, and methodology used for Ki-67 assessment [15, 16]. For example, Poggi et al. demonstrated that dogs with intermediate Ki-67 expression (20.1–40%) had longer lymphoma-specific survival and relapse-free intervals than dogs with either low (≤ 20%) or high (> 40%) Ki-67 expression in high-grade canine B-cell lymphoma treated with a modified Wisconsin–Madison protocol [15]. In the present case, the Ki-67 labeling index was relatively low (14.28%), and mitotic figures were infrequent (0–3 per ×400 microscopic field), suggesting relatively limited proliferative activity. Although Ki-67 expression is generally correlated with mitotic activity in canine lymphoma, a low Ki-67 index does not necessarily indicate favorable biological behavior or prognosis. Despite the relatively low proliferative index, the tumor showed rapid clinical progression and poor therapeutic response, indicating that proliferative activity alone may not fully reflect the biological behavior of this neoplasm.

In dogs, lymphoma immunophenotypes and clonality can be reliably determined using a combination of IHC, flow cytometry, and PARR [10, 18]. In the present case, flow cytometry analysis of the cutaneous lesion demonstrated a predominance of CD79a-positive B cells with few CD3-positive T lymphocytes, consistent with a B-cell-dominant neoplastic population accompanied by reactive T cells. PARR analysis confirmed the clonal rearrangement of the immunoglobulin heavy chain gene, establishing B-cell clonality and excluding reactive or inflammatory lymphoid processes. Together, histopathology, IHC, flow cytometry, and PARR provided robust complementary evidence that cutaneous lesions represented true neoplastic involvement of clonal B-cell lymphoma rather than a reactive lymphoid process.

Owing to the paucity of cases of cutaneous B-cell lymphoma, no standardized treatment regimen has been developed. Treatment options from the literature include the COP-based protocol, CHOP-based protocol, prednisolone alone, and doxorubicin with prednisolone after en bloc tumor resection [4, 5, 8, 9]. Treatment responses varied. One dog achieved complete remission, and no recurrence occurred for 3 years after gross tumor resection and chemotherapy with doxorubicin and prednisolone [9]. In contrast, one dog received a CHOP-based protocol and showed an initial response; however, the dog deteriorated quickly, and the owner elected euthanasia after the initiation of treatment, highlighting the potential aggressiveness of this tumor [8]. Similarly, this patient showed an initial partial response to CHOP-based multi-agent chemotherapy, followed by rapid disease progression and a nondurable response. One dog achieved complete remission with a COP-based protocol but relapsed after 120 days [5]. In canine cutaneous T-cell lymphoma, lomustine-based chemotherapy (lomustine, L-asparaginase, and prednisolone) achieved a 67% response rate; however, the median progression-free interval of 60 days was undesirable [19]. Although several treatment options have been considered, the optimal treatment regimen for canine non-epitheliotropic cutaneous B-cell lymphoma remains unclear. In canine cutaneous T-cell lymphoma, histopathological subtype, presence of neoplastic lymphocytes in the peripheral blood, thrombocytopenia, and initial response to chemotherapy were identified as significant prognostic factors. Dogs with epitheliotropic cutaneous T-cell lymphoma have a significantly shorter overall survival time than those with non-epitheliotropic cutaneous T-cell lymphoma [19]. However, due to the low incidence of cutaneous B-cell lymphoma, studies investigating its prognostic factors are lacking.

As discussed above, strong and diffuse MUM-1 expression suggests an activated or post–germinal center B-cell phenotype, whereas a relatively low Ki-67 labeling index and low mitotic activity suggest limited proliferative activity. Although the biological and prognostic significance of MUM-1 expression in canine lymphoma has not yet been fully established [11], MUM-1 expression has been associated with biologically aggressive behavior and poor clinical outcomes in human lymphoma, and similar findings have been reported in a pilot study of canine high-grade B-cell lymphoma [11–14]. Although no universally accepted cutoff value for Ki-67 expression has been established for canine lymphoma, previous studies have suggested that dogs with intermediate Ki-67 expression may have better clinical outcomes than those with either low or high Ki-67 indices [15]. These findings indicate that low Ki-67 expression does not necessarily predict favorable biological behaviors. In this case, despite the relatively low Ki-67 labeling index and infrequent mitotic figures, the tumor showed rapid clinical progression and a poor therapeutic response. Although the prognostic significance of MUM-1 and Ki-67 has not been clearly established in canine cutaneous B-cell lymphoma because of its rarity, the strong MUM-1 expression observed in the present case, despite the relatively low proliferative index, may suggest an aggressive biological phenotype associated with rapid disease progression and an incomplete response to CHOP-based chemotherapy. Further studies are needed to clarify the prognostic and therapeutic relevance of these biomarkers in canine non-epitheliotropic cutaneous B-cell lymphomas.

This study has several limitations inherent to a single-case study. First, although imaging showed mild lymph node enlargement, lymphoma involvement in the peripheral and abdominal lymph nodes was not confirmed by histopathological evaluation or additional diagnostic assessment. Second, imaging analysis indicated hepatomegaly, and hepatic fine-needle aspiration revealed rare atypical lymphoid cells. However, their low frequency precluded definitive cytological confirmation of hepatic lymphoma infiltration, and no further ancillary diagnostic tests, such as histopathological examination or PARR of cytological samples, were performed to achieve a definitive diagnosis. Finally, postmortem examination was not available, precluding a thorough assessment of the extent of systemic disease. Taken together, imaging, molecular (PARR), and cytological findings showed no definitive evidence of systemic lymphoma, thereby clearly demonstrating multifocal cutaneous involvement by clonal B-cell lymphoma. However, subclinical or early systemic involvement could not be completely excluded because postmortem examination was not performed.

In conclusion, this report describes a rare case of multifocal non-epitheliotrophic cutaneous B-cell lymphoma in a dog, characterized by aggressive biological behavior, high proliferative activity, and poor response to conventional chemotherapy. Given the paucity of comparable cases in veterinary literature, this case provides valuable clinicopathological, immunophenotypic, and molecular data and highlights the need for further studies to better define the classification, biological behavior, and optimal management of this uncommon lymphoma subtype.

## Supplementary Information


Supplementary Material 1.


## Data Availability

Data used in this study are available from the corresponding author upon request.

## Abbreviations

CD: Cluster of Differentiation
PAX5: Paired Box 5
MUM-1: Multiple Myeloma Oncogene 1
Ki-67: Marker of proliferation Ki-67
PARR: Polymerase Chain Reaction for Antigen Receptor Rearrangement
WHO: World Health Organization
ALT: Alanine Aminotransferase
ALP: Alkaline Phosphatase
GGT: Gamma-Glutamyl Transferase
BUN: Blood urea nitrogen
CT: Computed Tomography
CHOP: Cyclophosphamide, Doxorubicin, Vincristine, and Prednisolone
PCR: Polymerase Chain Reaction
IgH: Immunoglobulin Heavy Chain
DLBCL: Diffuse Large B-Cell Lymphoma
ABC: Activated B-Cell
FITC: Fluorescein Isothiocyanate
PE: Phycoerythrin
IHC: Immunohistochemistry
