# Supplementary material for: Multifocal non-epitheliotrophic cutaneous B-cell Lymphoma in a dog: cytologic, histopathologic, immunophenotypic, and molecular findings
Source: BMC Vet Res. 2026 Jun 20;22:510. doi: 10.1186/s12917-026-05655-w (PMC13536579; doi:10.1186/s12917-026-05655-w)
Supplement: Supplementary file 1 [file 12917_2026_5655_MOESM1_ESM.pdf]

Supplementary Information

Multifocal Cutaneous B-Cell Lymphoma in a Dog: Cytologic, Histopathologic, Immunophenotypic, and Molecular Findings

Yunho Jeong[1,2], Min-Ji Lee[1], Sooyoung Choi[3], Dae-Yong Kim[4], Jin-Young Chung[1], Jin-Ok Ahn[1],*, Eun Wha Choi[5],*

[1]Department of Veterinary Internal Medicine, College of Veterinary Medicine, Kangwon National University, 1 Kangwondaehak-gil, Chuncheon, Gangwon-do, 24341, Republic of Korea

[2]Haemaru Companion Animal Clinical Research Institute, 8-4, Hwangsaeul-ro 319beon-gil, Bundang-gu, Seongnam-si, Gyeonggi-do, 13590, Republic of Korea

[3]Department of Veterinary Medical Imaging, College of Veterinary Medicine & Institute of Veterinary Science, Kangwon National University, 1 Kangwondaehak-gil, Chuncheon, Gangwon-do, 24341, Republic of Korea

[4]Department of Veterinary Pathology and Research Institute of Science, College of Veterinary Medicine, Seoul National University, 599 Gwanak-ro, Gwanak-gu, Seoul, 08826, Republic of Korea.

[5]Department of Veterinary Clinical Pathology, College of Veterinary Medicine & Institute of Veterinary Science, Kangwon National University, 1 Kangwondaehak-gil, Chuncheon, Gangwon-do, 24341, Republic of Korea

***Corresponding author:**

Jin-Ok Ahn, DVM, PhD, Associate professor

Department of Veterinary Internal Medicine, College of Veterinary Medicine, Kangwon National University, 1 Kangwondaehak-gil, Chuncheon, Gangwon-do, 24341, Republic of Korea

Phone: 82-33-250-8790

Fax: 82-33-259-5625

E-mail: joahn@kangwon.ac.kr

***Corresponding author:**

Eun Wha Choi, DVM, PhD, Associate professor

Department of Veterinary Clinical Pathology, College of Veterinary Medicine & Institute of Veterinary Science, Kangwon National University, 1 Kangwondaehak-gil, Chuncheon, Gangwon-do, 24341, Republic of Korea

Phone: 82-33-250-8794

Fax: 82-33-259-5625

E-mail: ewchoi@kangwon.ac.kr

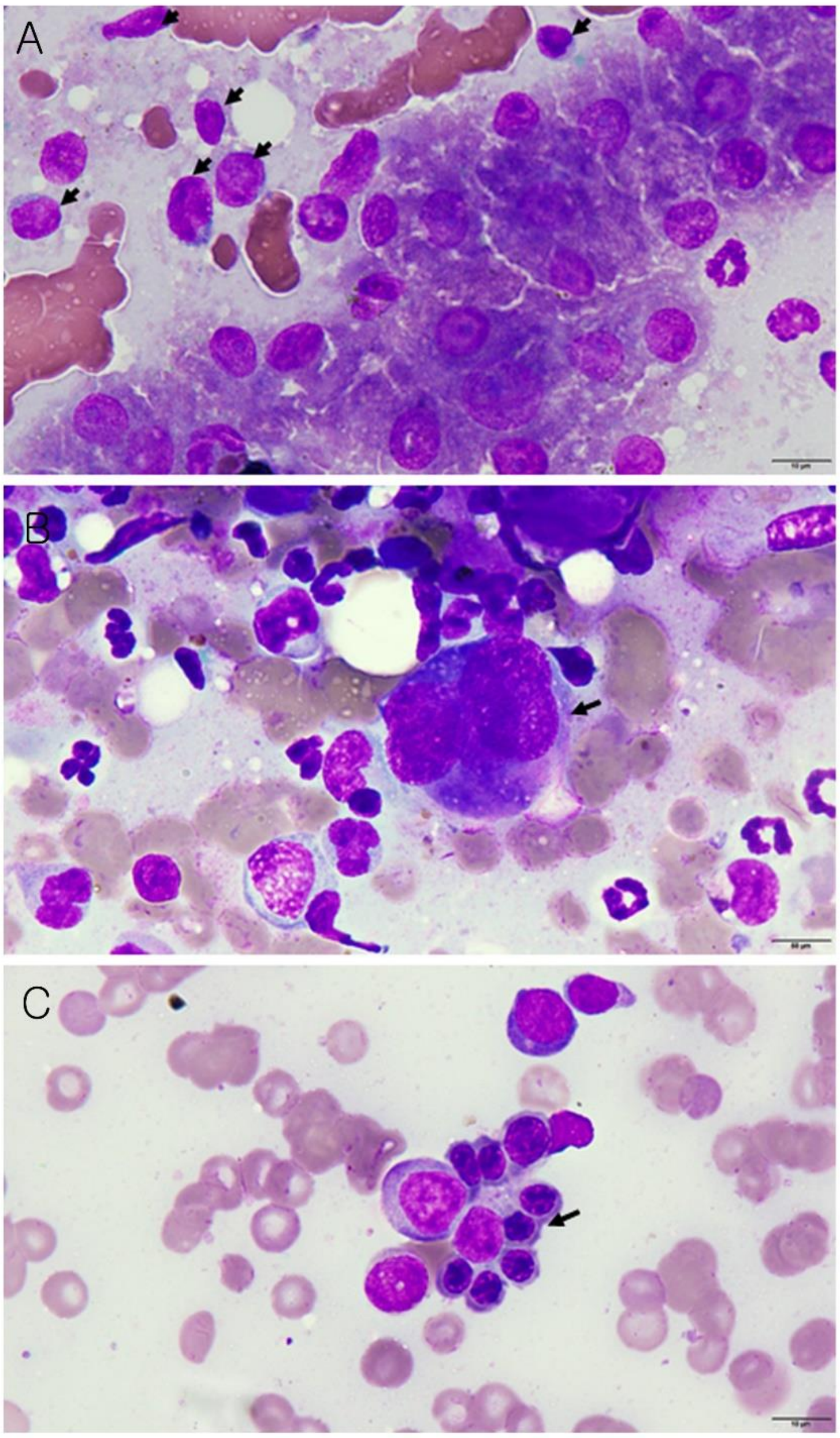

Supplementary Figure 1. Cytologic findings from liver and spleen fine-needle aspirates.

(A) Liver aspirate showing scattered small, mature lymphocytes (arrows) characterized by a high nuclear-to-cytoplasmic ratio and densely clumped chromatin, admixed with hepatocytes.

(B) Splenic aspirate demonstrating a large megakaryocyte (arrow) with abundant cytoplasm and a multilobulated nucleus, consistent with extramedullary hematopoiesis.

(C) Splenic aspirate showing nucleated red blood cells (arrow), with round nuclei and variably basophilic cytoplasm, also consistent with extramedullary hematopoiesis.

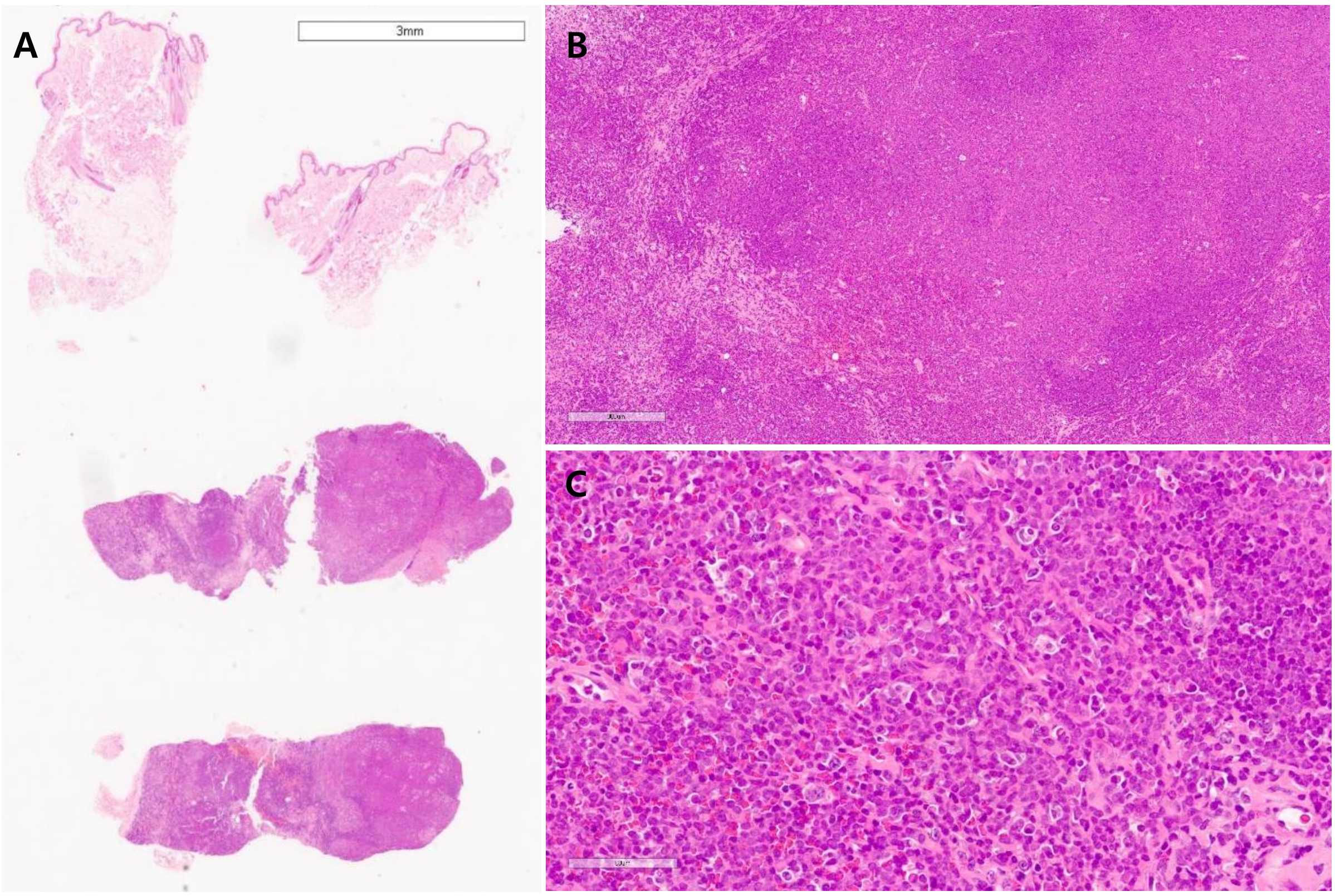

Supplementary Figure 2. Photomicrographs of the cutaneous mass at different magnifications. (A) Very low-power view showing a dermal/subcutaneous mass separated from the epidermis without evidence of epitheliotropism, supporting a non-epitheliotropic pattern (scale bar = 3 mm). (B) Low-power view showing densely cellular neoplastic round-cell proliferation involving the dermis and subcutis (scale bar = 300 μm). (C) High-power view showing that the neoplastic population is composed predominantly of lymphoid cells with a high nuclear-to-cytoplasmic ratio and scant cytoplasm. Scattered plasma cells, neutrophils, and eosinophils are admixed within the lesion (scale bar = 60 μm).
